# Transcriptome Analysis and Identification of Differentially Expressed Transcripts of Immune-Related Genes in Spleen of Gosling and Adult Goose

**DOI:** 10.3390/ijms160922904

**Published:** 2015-09-22

**Authors:** Anqi Wang, Fei Liu, Shun Chen, Mingshu Wang, Renyong Jia, Dekang Zhu, Mafeng Liu, Kunfeng Sun, Ying Wu, Xiaoyue Chen, Anchun Cheng

**Affiliations:** 1 Institute for Preventive Veterinary Medicine, Sichuan Agricultural University, Chengdu 611130, China; E-Mails: anqiwang77@163.com (A.W.); mshwang@163.com (M.W.); cqrc_jry@163.com (R.J.); liumafengra@163.com (M.L.); sunkunfeng1981@163.com (K.S.); yingzi_no1@126.com (Y.W.); 2 Key Laboratory of Animal Disease and Human Health of Sichuan Province, Sichuan Agricultural University, Chengdu 611130, China; E-Mails: liufei@sicau.edu.cn (F.L.); zdk24@163.com (D.Z.); sophia_cs@yeah.net (X.C.); 3 Avian Disease Research Center, College of Veterinary Medicine of Sichuan Agricultural University, Chengdu 611130, China

**Keywords:** gosling, adult goose, immune molecular development, comparative transcriptomics

## Abstract

The goose (*Anser cygnoides*), having high nutritional value, high-quality feathers and high economic benefit, is an economically important poultry species. However, the molecular mechanisms underlying the higher susceptibility to pathogens in goslings than in adult geese remains poorly understood. In this study, the histological sections of spleen tissue from a two-week-old gosling and an adult goose, respectively, were subjected to comparative analysis. The spleen of gosling was mainly composed of mesenchyma, accompanied by scattered lymphocytes, whereas the spleen parenchyma was well developed in the adult goose. To investigate goose immune-related genes, we performed deep transcriptome and gene expression analyses of the spleen samples using paired-end sequencing technology (Illumina). In total, 50,390 unigenes were assembled using Trinity software and TGICL software. Moreover, these assembled unigenes were annotated with gene descriptions and gene ontology (GO) analysis was performed. Through Kyoto encyclopedia of genes and genomes (KEGG) analysis, we investigated 558 important immune-relevant unigenes and 23 predicted cytokines. In addition, 22 immune-related genes with differential expression between gosling and adult goose were identified, among which the three genes showing largest differences in expression were immunoglobulin alpha heavy chain (*IgH*), mannan-binding lectin serine protease 1 isoform X1 (*MASP1*) and C–X–C chemokine receptor type 4 (*CXCR4*). Finally, of these 22 differentially expressed immune-related genes, seven genes, including tumor necrosis factor receptor superfamily member 13B (*TNFRSF13B*), C-C motif chemokine 4-like (*CCL4*), *CXCR4*, interleukin 2 receptor alpha (*IL2RA*), *MHC* class I heavy chain (*MHCIα*), transporter of antigen processing 2 (*TAP2*) *IgH*, were confirmed by quantitative real-time PCR (qRT-PCR). The expression levels of all the candidate unigenes were up-regulated in adult geese other than that of *TNFRSF13B*. The comparative analysis of the spleen transcriptomes of gosling and adult goose may promote better understanding of immune molecular development in goose.

## 1. Introduction

The goose (*Anas cygnoides*), an economically important poultry species, is cultivated widely in China which has become the country breeding the most geese in the world. Compared with other avian terrestrials, the waterfowl shows asymptomatic and long-lasting infection when infected with avian viruses, such as the avian influenza virus. Once an avian virus invades host cells, the first steps of the cellular defense mechanisms involve the sensing of pathogen-associated molecular patterns (PAMPs) by pattern recognition receptors (PRRs), including retinoic acid-inducible gene I (RIG-I)-like receptors (RLRs), Toll-like receptors (TLRs) and the nucleotide oligomerization domain (NOD)-like receptors (NLRs). Then interferon (IFN) signaling is activated, inducing the production of IFN-stimulated genes (ISGs), which can inhibit virus replication.

A thorough understanding of the waterfowl immune system will help us better understand the molecular mechanisms of the host-pathogen interaction. As a potential transmitter of avian viruses, goose can provide an invaluable model for studies on waterfowl immunology. Moreover, goslings have a low ability to resist disease compared to adult geese because goslings have an underdeveloped immune system. However, the molecular basis of the goose immune system is relatively little. Here, to better understand immune development in goose, immune-related genes with differential expression between gosling and adult goose were identified. Besides, the discovery of immune-relevant genes may facilitate the elucidation of immunological responses.

Over the past decade, next-generation sequencing technology has emerged as a revolutionary tool for transcriptomics [1]. With the advantages of speed, precision and high-efficiency performance, this success has encouraged us to explore the uncharacterized non-model species and to discover diversities among individuals [2,3]. To date, next-generation sequencing has been applied in various animals, including geese. At present, goose transcriptome profiling has mainly been based on ovarian tissues [4,5], mixed tissues [6], exocrine tissue [7], and hepatic tissue [8], and the identified genes are responsible for reproductive biology, development and metabolism processes, as well as laying performance and fatty liver breeding, respectively. Intriguingly, next generation sequencing has proved to be an effective approach for discovering genes that are associated with the immune response [3]. Recently, the transcriptomic analysis of goose peripheral blood lymphocytes was conducted to identify immune genes [9]. Nevertheless, no studies have characterized gene expression in the goose spleen, an immune organ that plays an important role in goose humoral immunity and cellular immunity [10]. Moreover, it has been reported that bird spleen acts as a primary site for lymphocyte antigen-independent differentiation and proliferation, unlike the mammalian spleen [11].

Considering the importance of the spleen in birds, we selected spleens as target samples to conduct histological and deep transcriptome analyses in both gosling and adult goose. A large number of undiscovered genes of goose were identified. Meanwhile, 558 unigenes, which may participate in the immune response, were identified, among which 15 unigenes were up-regulated and seven unigenes were down regulated in adult goose spleen compared to gosling spleen. These differentially expressed genes may be closely related to the development of goose immune molecules. Moreover, our study identified 23 cytokines, which may be useful in goose vaccine development and also can shed lights on the study of the cytokine signal pathway in goose. Overall, the transcriptome datasets provide a valuable resource for the investigation of the goose immune system.

## 2. Results and Discussion

### 2.1. Histological Analysis

**Figure 1:**
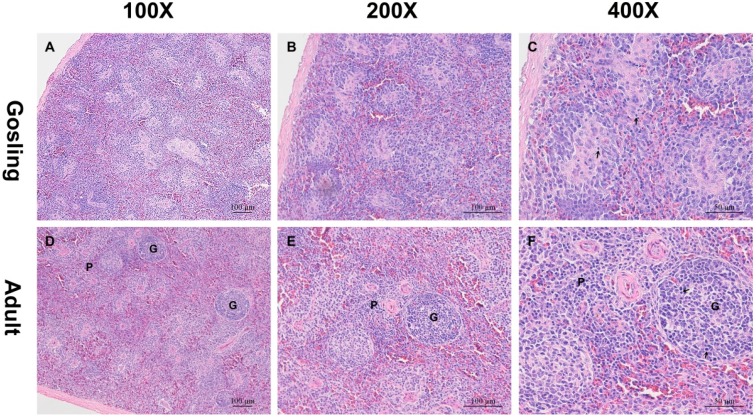
Histological changes in goose spleens at different developmental stages. The spleen samples from the gosling (**A**–**C**), and the adult goose (**D**–**F**) were cut into sections and stained with hematoxylin and eosin (H&E). The parenchyma in the gosling spleen was sparse. However, well-developed spleen parenchyma was detected in the adult goose. G indicates germinal centers; P indicates periarteriolar lymphoid sheaths; arrows indicate lymphocytes.

In goose, the spleen is the largest lymphoid organ and consists of red and white pulp. The main element of the former is the supporting blood-filled sinusoid, and the latter is predominantly populated by lymphoid tissue. However, there is not a distinct boundary between them, particularly in the spleen of the gosling. In the gosling spleen, the mesenchyma was abundant, but the spleen parenchyma was relatively sparse (Figure 1A–C). Instead of a distinct white pulp, scattered lymphocytes could be detected.

However, the spleen of the adult goose differed in structure from that of the gosling. The adult spleen parenchyma was well developed and replaced the mesenchyma. The splenic nodules were distinct, surrounded by connective tissue and containing relatively large germinal centers (GCs). The periarteriolar lymphoid sheaths (PALS) were developed with lymphocytes (Figure 1D–F). Because gosling spleen is immature, all of the above features were absent. Bird spleen acts as a primary site for lymphocyte antigen-independent differentiation and proliferation. Therefore, gosling spleens do not contain sufficient mature lymphocytes to resist pathogens.

### 2.2. Illumina Sequencing and de Novo Assembly

After sequencing using Illumina HiSeqTM 2000 system, we generated 63,906,458 and 69,459,974 raw reads in the gosling and goose libraries, respectively. After removal of low-quality reads and reads containing adaptor or N (It means the bases in reads were not certain), the libraries yielded totals of 63,121,772 and 68,655,944 clean reads. Their Q30 values (meaning that the base recognition accuracy is 99.9%) were 93.18% and 93.25%, respectively (Table 1). Trinity software [12] was used to assemble the clean reads, producing 180,210 assembled sequences with a total length of 181,908,092, an average length of 1009.42 and an N50 of 1725. After clustered by TGICL [13], a total of 50,390 unigenes with a total length of 79,599,367, an average length of 1579.67 and an N50 of 2585 were obtained (Table 1). The sequencing raw data have been deposited into the Short Reads Archive (SRA) database under the accession number SRR2125737.

**Figure 2:**
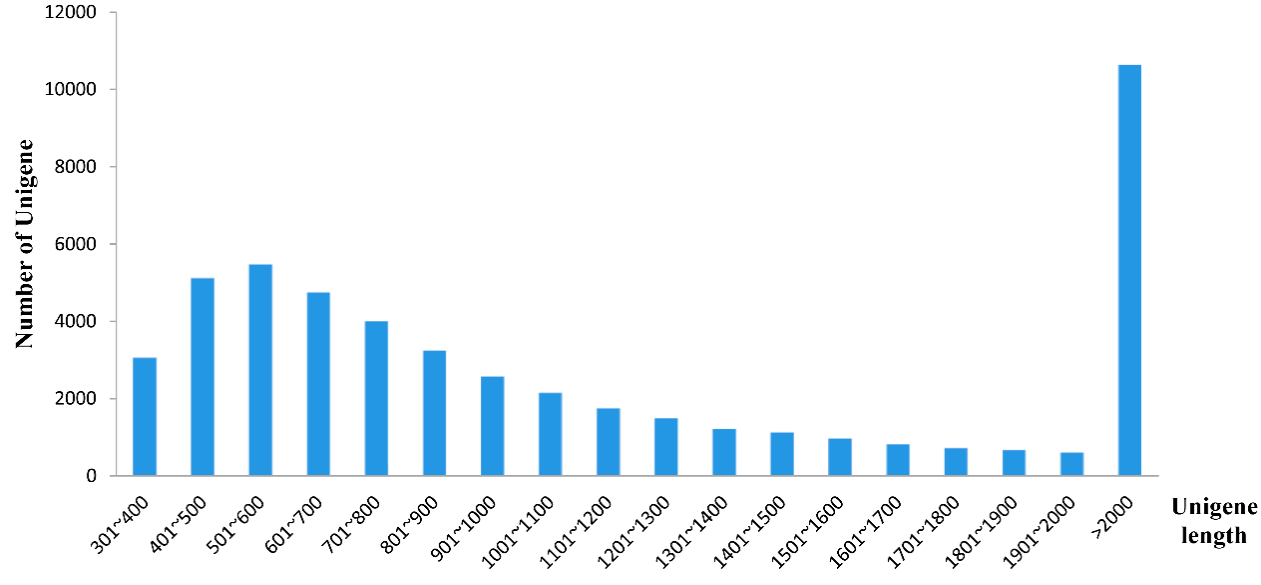
Histogram of the unigene length distribution. The *x*-axis indicates the length range of unigenes. The *y*-axis denotes the number of unigenes in every range of length.

All assembled unigenes were more than 300 bp in size and the maximum length of unigenes was 31,052 bp. Among these unigenes, 28,205 unigenes (55.97%) were in the length range of 300 to 1000, 11,567 unigenes (22.96%) were in the length range of 1000 to 2000 and 10,618 unigenes (21.07%) were longer than 2000 bp (Figure 2). Compared with previous goose transcriptome databases [4,6,9], the average length and the N50 of unigenes in our study were much longer. In addition, nearly half of these unigenes were more than 1000 bp in size. All of the data indicated that the integrity of unigenes was relatively good.

**Table 1:** Summary for goose spleen transcriptome.

Description	2 Week-Gosling	Adult Goose	Both
RIN (RNA integrity Number)	10.0	10.0	
Raw reads	63,906,458	69,459,974	
Clean reads	63,121,772	68,655,944	
Q30 (Q-score)	93.18%	93.25%	
Total number of unigenes			50,390
Total length of unigene			79,599,367
Max length of unigene			31,052
Min length of unigene			301
Average length of unigene			1579.67
N50			2585

### 2.3. Assembly Evaluation and Annotation

**Figure 3:**
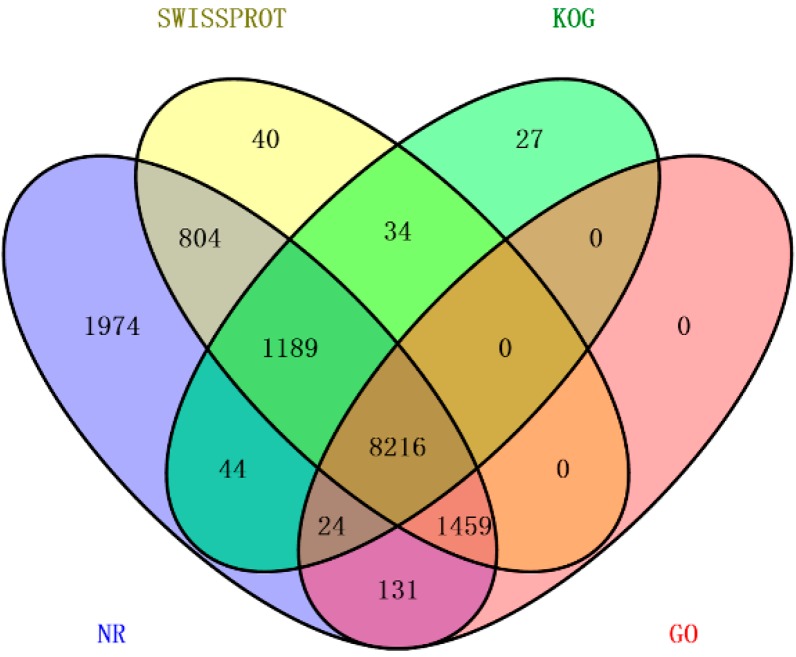
Comparison of the number of unigene annotations obtained from the different databases. Each section illustrates the number of unigenes shared among the Nr, Swiss-Prot, KOG and GO databases.

**Figure 4:**
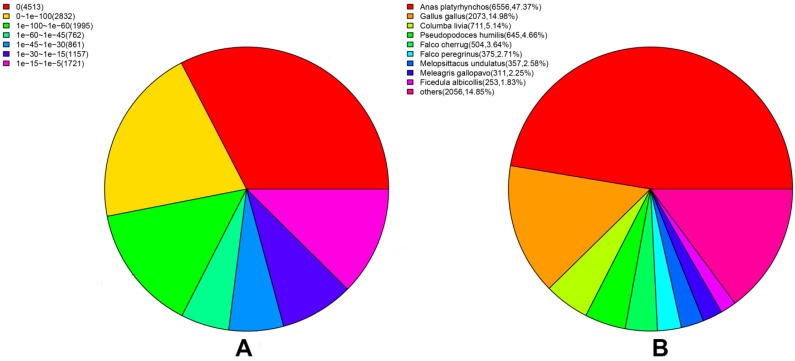
Statistical analysis of the assembled unigenes against Nr database. (**A**) *E*-value distribution; (**B**) Species distribution.

Through the annotation of unigenes, out of 50,390 unigenes, 27.47%, 23.30%, 19.51%, 18.92% and 7.16% unigenes received significant hits in the non-redundant (Nr), Swiss-Prot, gene ontology (GO), clusters of orthologous groups for eukaryotic complete genomes (KOG) and Kyoto encyclopedia of genes and genomes (KEGG) databases, respectively (Table 2). With the help of the online software Venny [14], the numbers of unigenes that were either unique or shared among databases were visualized with a Venn diagram (Figure 3). Among the 50,390 unigenes, 8216 unigenes had reference sequences in all four databases and 13,942 (27.37%) unigenes were aligned to at least one database. However, 36,448 (72.33%) unigenes could not be annotated, likely because these unigenes may include a large number of novel genes or non-coding RNA sequences. Besides, these non-annotated unigenes might be too short to generate sequence matches. We also noticed that 13,841 unigenes could be correctly annotated in the Nr database. The *E*-value distribution of unigenes mapped to the Nr database showed that 16.04% of the unigenes had perfect matches, 42.45% of the unigenes showed significant homology to the stored sequences, and 41.51% of the unigenes showed homology ranging from 1 × 10^−45^ to 1 × 10^−5^. In addition, the species distribution indicated that at least 80.44% of the unigenes matched those of avian species. Moreover, 43.53% of the transcripts had the highest homology with *Anas platyrhynchos*, followed by *Gallus gallus* (14.83%) and *Columba livia* (7.89%) (Figure 4). The result was consistent with the genetic relationship between these species, thus supporting the validity of our transcriptome data.

**Table 2:** Annotation of unigenes BLAST against five different databases.

Database	Number of Annotated Unigenes	Annotated Unigene Ratio (%)
Nr	13,841	27.47%
Swiss-Prot	11,742	23.30%
GO	9830	19.51%
KOG	9534	18.92%
KEGG	3607	7.16%
unknown	36,448	72.33%

### 2.4. Function Classification and Pathway Analysis

#### 2.4.1. KOG Analysis

**Figure 5:**
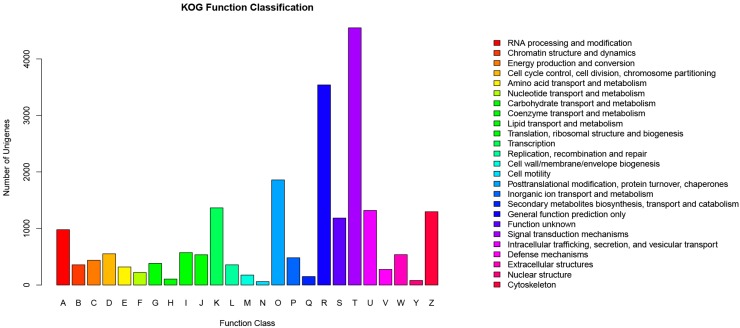
Histogram presentation of eukaryotic clusters of orthologous groups (KOG) classification. A total of 9534 sequences were clustered into 25 KOG categories.

Using the KOG databases, we predicted the function of the unigenes and classified them according to possible functions. Among the 25 KOG categories, the cluster for “Signal transduction mechanisms” represented the largest group (4551, 47.73%), followed by “General function prediction only” (3540, 37.13%) and “Posttranslational modification, protein turnover, chaperones” (1858, 19.49%). The smallest groups were “Nuclear structure” (80, 0.84%) and “Cell motility” (60, 0.63%). Figure 5 also shows that 276 unigenes were involved in the cluster for “Defense mechanisms”. In this experiment, the largest group was “Signal transduction mechanisms”, whereas in many other studies, the cluster for “General function prediction only” was identified as the largest group [4,15,16]. These results indicate signal transduction mechanisms may be more important for goose splenic tissue than for other tissues. With this in mind, we paid more attention to the signal transduction mechanisms. Furthermore, many of the unigenes participated in defense mechanisms, which may imply that the spleen is an important peripheral immune organ.

#### 2.4.2. GO Analysis

**Figure 6:**
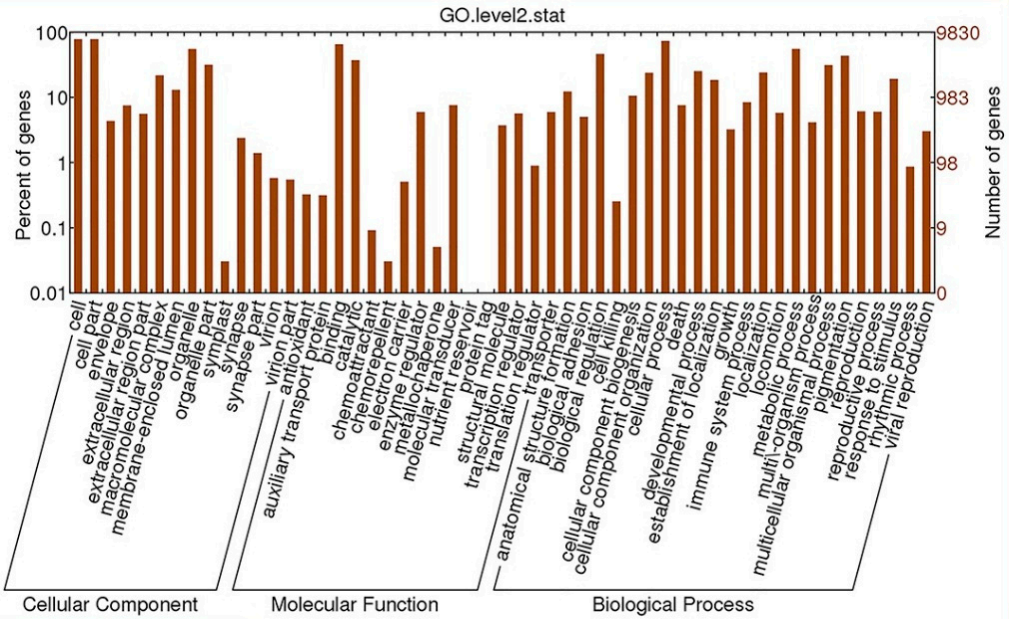
Histogram presentation of Gene Ontology classification. Unigenes were assigned to three categories: cellular components, molecular functions and biological processes.

Using Blast2GO software, we classified these sequences at two levels. Finally, 9830 transcripts were divided into three main categories (cellular component, molecular function and biological process) and 64 subcategories. In the subcategories of cellular components, most of the corresponding genes were involved in “cell” (7042 unigenes), “cell part” (7042 unigenes) and “organelle” (5558 unigenes) (Figure 6). In the categories of molecular function, the largest term is “binding” into which 6397 unigenes were classified, followed by the terms “catalytic activity” (3686 unigenes) and “molecular transducer activity” (612 unigenes). However, we only find one gene each in the “nutrient reservoir” and “protein tag” clusters. The biological process category, assigned 4414 unigenes, held the majority of genes. We also observed that 857 and 3439 unigenes belonged to the categories “immune system process” and “response to stimulus”, respectively. It is noteworthy that in GO analysis, one unigene can be classified into more than one GO term due to its multiple functions. Hence, these unigenes in the cluster “immune system process”, likely also belonged to the “response to stimulus” catergory. Nevertheless, among the 9830 transcripts that underwent GO analysis, at least 3439 unigenes (approximately 35%) participated in stress or defense response. However, only 0.3% and 2.8% unigenes were divided into in the cluster of “immune system process” and “response to stimulus”, respectively [9]. The spleen, which was chosen for our study, is an important site of resistance or defense response, which may explain the high proportion of unigenes involved in resistance or defense system.

#### 2.4.3. KEGG Analysis

**Figure 7:**
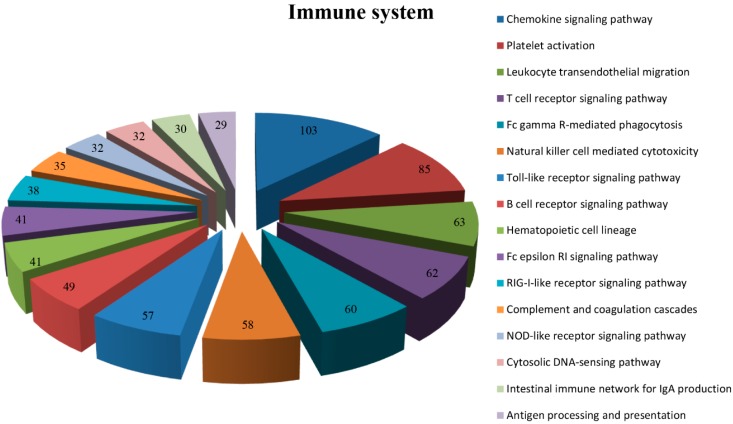
Distribution of unigenes in the immune system. In the immune system, 16 pathways were identified. A total of 456 unigenes were involved in the 16 immune system KEGG pathways. Each unigene may be grouped into more than one pathway.

For KEGG analysis, unigenes were searched against the KEGG database to identify biological processes in which they participated. Here, a total of 3607 unigenes were assigned to 332 KEGG pathways. The top three pathways with most representation of unigenes were “Pathways in cancer” (198 unigenes), “PI3K–Akt signaling pathway” (183 unigenes) and “HTLV-I infection” (159 unigenes). In the past, much research has been conducted to reveal the mechanism of cancer and the process of carcinogenesis, and cancer is a complicated process that involves many genes. Therefore, many genes involved in cancer pathway were identified. Moreover, the relationship between cancer and the PI3K–Akt signaling pathway has been highlighted [17] and the PI3K–Akt signaling pathway was involved in numerous cellular processes such as metabolism, inflammation, cell survival, motility, and cancer progression [18]. Thus, it is unsurprising that the two largest pathway groups were “Pathways in cancer” and the “PI3K–Akt signaling pathway”. Human T-cell lymphotrophic virus 1 (HTLV-I) is a disease closely related to the immune system. The unigenes clustered for “HTLV-I infection” might be relevant to immunoreactions, reflecting the role of the spleen in the immune system. Meanwhile, the finding that the top three pathway included “Pathways in cancer” and “HTLV-I infection” coincided with reports by other researchers [6]. Nevertheless, we were most interested in the 16 KEGG pathways in the “immune system” group. As shown in Figure 7, the three pathways with the largest number of unigenes were “Chemokine signaling pathway” (103 unigenes), “Platelet activation” (85 unigenes), and “Leukocyte transendothelial migration” (63 unigenes). In addition, some signaling pathways associated with the immune response were not classified into the “immune system” group, such as “Cytokine–cytokine receptor interaction”, in which 108 unigenes were identified. With so many unigenes participating in “Cytokine–cytokine receptor interaction” pathway and “Chemokine signaling pathway” representing the largest group in the “immune system” category, we speculated that cytokines play an important role in signal transduction and immune responses in spleen. The large number of unigenes identified as related to cytokine signaling pathways may also be because the cytokine family is composed of many members.

It is noteworthy that 23 unigenes encoding cytokines were identified (Table 3). The first cytokine was originally described in 1957 in chick embryo [19], but the number of identified avian cytokines and the knowledge of their characteristic and potential applications have remained limited because they are not well conserved among species [20]. Fortunately, new technology and high-throughput sequencing quickly changed this situation. Many avian cytokines have now been identified, and their potential applications have been investigated, particularly their potential roles as therapeutic agents or vaccine adjuvants [21,22]. In this study, we identified 23 unigenes annotated as cytokines or chemokines. Cytokines are soluble proteins secreted by a broad range of cell types, and they are regarded as extracellular signals between cells during the course of immune responses [23]; chemokines are a special type of cytokine with a role in leucocyte chemotaxis. In general, cytokines are divided into six groups: the interleukins (IL), the interferons, the transforming growth factor-β (TGF-β) family, the tumour necrosis factor (TNF) superfamily (TNFSF), the colony-stimulating factors (CSF) and the chemokines.

In the interleukins family, we identified three genes: *ILz1β*, *IL6* and *IL18*. *IL6* is a multifunction cytokine. Its immune function includes the differentiation of B cells to plasma cells, T-cell growth and differentiation, the differentiation myeloid leukemic cell lines into macrophages, and megakaryocyte maturation [24]. Both *IL1β* and *IL18* are members of the *IL1* family, and they are co-stimulators of T cell functions. As the *IFN*-γ-inducing factor, *IL18* in combination with *IL2*, *IL12*, or *IL15* can stimulate *IFN*-γ with the ability to induce cellular resistance to viral pathogens. Moreover, *IL1β* has been reported to play an essential role in T-cell-dependent antibody production [25]. The function of *IL1β* as a growth factor for B cell proliferation was also verified, possibly due to *IL1*-mediated induction of *IL6* [26].

**Table 3:** A list of unigenes belonging to the cytokine family.

Group Name	Unigene ID	Cytokine	Gene Description	Human	Chicken	Duck	Goose	Identity
Interleukin	CL32153Contig1	*IL6*	interleukin 6	M54894.1	NM_204628.1	XM_005020542.1	JF437643.1	73%
	CL35348Contig1	*IL1B*	interleukin 1 beta	M15330.1	NM_204524.1	Absent	JF505290.1	93%
	CL30075Contig1	*IL18*, *IL1F4*	interleukin 18	AY044641.1	NM_204608.1	DQ522948.1	JF505289.1	99%
Interferon	CL30945Contig1	*IFNA*	interferon alpha	M54886.1	EU367971.1	KF731866.1	HQ1155831	88%
Tumor necrosis factor	CL13660Contig1	*TNFSF4*, *OX40L*	tumor necrosis factor ligand	NM_003326.4	XM_430147.3	Absent	Absent	94%
			superfamily member 4	NM_001297562.1				
	CL33231Contig1	*TNFSF5*, *CD40L*	tumor necrosis factor ligand	AY197739.1	AJ293700.1	DQ267671.2	Absent	99%
			superfamily member 5					
	CL36855Contig1	*TNFSF6*, *FASL*	tumor necrosis factor ligand	AY225406.1	AJ890143.1	Absent	Absent	99%
			superfamily member 6					
	CL36714Contig1	*TNFSF10*, *TRAIL*	tumor necrosis factor ligand	NM_003810.3 NM_001190942.1 NM_001190943.1	NM_204379.2	XM_005017465.1	Absent	99%
			superfamily member 10			XM_005017466.1		
	CL44515Contig1	*TNFSF11*, *RANKL*	tumor necrosis factor ligand	NM_003701.3 NM_033012.3	NM_001083361.1	XM_005008959.1	Absent	85%
			superfamily member 11					
	CL14930Contig1	*TNFSF15*, *VEGI*	tumor necrosis factor ligand	NM_005118.3 NM_001204344.1	NM_001024578.1	XM_005022362.1	Absent	92%
			superfamily member 15					
	CL448Contig2	*TNFSF13B*, *BAFF*	tumor necrosis factor ligand	NM_006573.4 NM_001145645.2	NM_204327.2	XM_005021732.1	DQ874394.1	99%
						XM_005021733.1		
			superfamily member 13B			XM_005021734.1		
						XM_005021735.1		
	CL34135Contig1	*EDA*	ectodysplasin-A	NM_001399.4	AY885699.1	XM_005019557.1	Absent	99%
				NM_001005609.1				
				NM_001005612.2				
				NM_001005610.3				
				NM_001005613.3				
Transforming growth factor	CL37958Contig1	*TGFB1*	transforming growth factor beta-1	NM_000660.5	JQ423909.1	Absent	Absent	83%
	CL35089Contig1	*TGFB2*	transforming growth factor beta-2	NM_001135599.2 NM_003238.3	NM_001031045.3	EU737316.1	EF541127.1	99%
	Unigene ID	*Cytokine*	*Gene Description*	Human	Chicken	Duck	Goose	Identity
Transforming growth factor	CL30050Contig1	*TGFB3*	*transforming growth factor beta-3*	NM_003239.3	NM_205454.1	XM_005013912.1	Absent	99%
Colony-stimulating factor	CL39251Contig1	*CSF1*, *MCSF*	*macrophage colony-stimulating factor 1*	NM_000757.5 NM_172210.2 NM_172211.3 NM_172212.2	GQ249403.1	XM_005015071.1	Absent	94%
Chemokine	CL34468Contig1	*CCL4*	*C-C motif chemokine 4*	NM_002984.3	NM_204720.1	Absent	Absent	99%
	CL38070Contig1	*CCL5*	*C-C motif chemokine 5*	NM_002985.2 NM_001278736.1	NM_001045832.1	Absent	Absent	99%
	CL35006Contig1	*CCL19*, *ELC*	*C-C motif chemokine 19*	NM_006274.2	NM_001302168.1	AY682098.1	Absent	100%
	CL39853Contig1	*CX3CL1*, *NTT*	*C-X3-C motif chemokine 1*	NM_002996.4 NM_001304392.1	NM_001077232.1	Absent	Absent	99%
	CL29319Contig1	*CXCL12*	*C–X–C motif chemokine 12*	NM_199168.3 NM_000609.6	NM_204510.1	XM_005029409.1XM_005029410.1	Absent	88%
				NM_001033886.2 NM_001178134.1				
				NM_001277990.11				
	CL19694Contig1	*CXCL13*	*C–X–C motif chemokine 13*	NM_006419.2	FR874037.1	Absent	Absent	99%
					FR874038.1			
	CL12315Contig1	*IL8*, *CXCL8*	*interleukin 8*	M28130.1	HM179639.1			

Accession numbers greater than 1 indicate transcript variants.

In the interferon group, only one member was identified, interferon alpha. *IFNα* was the first cytokine to be described [19] and has been well known as an antiviral factor. With the function of resistance to viruses, *IFNα* has been widely used in the prevention and treatment of diseases. It has also been demonstrated that several epidemic avian viruses including AIV, can be inhibited by chicken *IFNα* [22]. Moreover, it has been reported that the combined application of chicken *IFNα* and chicken *IL18* in a vaccine can effectively elevate immunomodulatory function [27]. These results may also be applicable to the development of goose vaccine.

In the *TNFSF*, *TGF-β* and *CSF* groups, eight, three and one unigene were discovered, respectively, most of which were not previously reported in goose. Both TNFSF and TGF-β are multifunction cytokines and they contribute not only to physiological processes but also pathological processes. Considering their role in the immune response, some of them have been applied to resistant avian diseases as molecular adjuvants [28,29].

Finally, seven unigenes were detected in the chemokines group. Based on the relative position of the first two conserved cysteines at the amino termini, the chemokines are divided into four subfamilies (XC, CC, CXC and CX3C). Though their roles in recruiting effector cells to infection sites have been demonstrated frequently, chemokines are also involve in many other processes, including the circulation of lymphoid cells. In the development of the spleen, C–X–C chemokine 13-like (*CXCL13*) can mediate the migration B cells towards the B cell follicles [30], whereas C–C motif chemokine 19-like (*CCL19*) and C–C motif chemokine 21-like (*CCL21*) are crucial for attracting T cells and dendritic cells (DCs) to the T cell zones of the white pulp [31].

Here, we also searched for the homologs of 23 unigenes in other species. We found that all 23 genes could be identified in human and chicken. However, the majority of the goose cytokines were not recorded in the National Center for Biotechnology Information (NCBI). In addition, the assembled sequences of these 23 unigenes were searched in the BlastN program and the identity with the highest matched sequence is listed in Table 3. Most identity levels of the 23 unigenes were at least 99%, which indicated the assembled sequences were predicted well. Here, these undiscovered cytokines of goose were identified, possibly providing insight into the complex networks regulated by cytokines in goose.

### 2.5. Analysis of Differentially Expressed Genes

**Figure 8:**
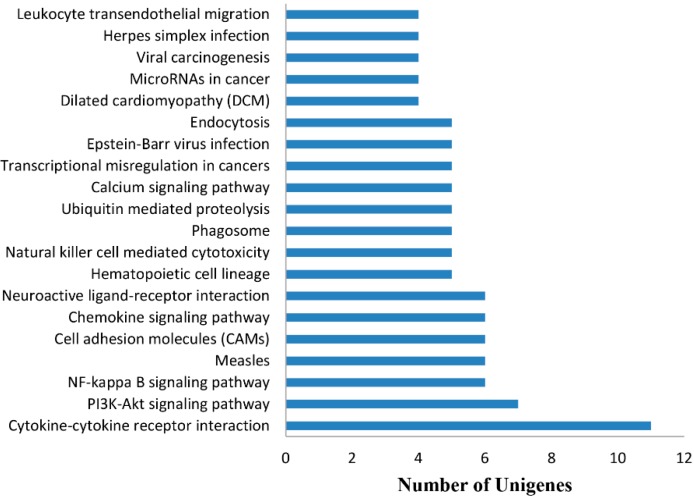
Top 20 KEGG pathways enriched up-regulated genes. Among the 1470 up-regulated unigenes, 11 unigenes are involved in the largest KEGG pathway “Cytokine–cytokine receptor interaction”. Five of the top 20 KEGG pathways participate directly in the immune response.

With Bowtie 2 [32] and eXpress [33], the number of reads mapped back to the orthologous region of each gene in proper pairs were 45,161,366 (71.54%) and 47,663,788 (69.42%) in the gosling sample and adult goose sample, respectively. More than half of the reads could map to each gene in proper pairs, which indicated that most reads were effective. With the false discovery rate (FDR) [34] method, in total, 2700 differentially expressed transcripts were detected (Table S2), with 1470 up-regulated and 1230 down-regulated genes. To understand which biological processes these differentially expressed genes were primarily associated with, the KEGG enrichment analysis of these genes was performed. Among KEGG pathways, the three pathways that contained the largest numbers of up-regulated unigenes were “cytokine–cytokine receptor interaction” (11 unigenes), “PI3K–Akt signaling pathway” (seven unigenes) and “NF-kappaB signaling pathway” (6 unigenes) (Figure 8, Table S3). These results indicated that many up-regulated genes were related to immune action. Moreover, there are two reasons why the cluster of “cytokine–cytokine receptor interaction” contained the largest number of up-regulated unigenes. First, the cytokine signaling pathway becomes more and more developed with the development of goslings; second, the number of unigenes classified as “Cytokine–cytokine receptor interaction” is larger than that of many other pathways. In addition, the KEGG pathways were sequentially arranged by the number of down-regulated unigenes that were mapped to these KEGG pathways (Table S4). Among the KEGG pathways enriched with down-regulated unigenes, we found the largest number of down-regulated unigenes were associated with the cell cycle pathway, likely because of the decline in the metabolism of adult goose.

Among the top 20 KEGG pathways enriched with up-regulated genes, five pathways directly participated in immune regulation: “cytokine–cytokine receptor interaction”, “chemokine signaling pathway”, “natural killer cell mediated cytotoxicity”, “phagosome” and “leukocyte transendothelial migration”. We therefore speculated that many changes occurred in immune gene expression during goose development.

### 2.6. Mining for Immunity Relevant Genes and Validation by Quantitative Real-Time PCR (qRT-PCR)

The spleen is an immune organ combining the innate immune system with the adaptive immune system [10]. Among these immune-related pathways, 558 important immune unigenes were identified and their detailed information is provided in Table S5; of thses unigenes, 22 immune-related unigenes were differentially expressed (Table 4). Among these differentially expressed genes, we screened for immune-related unigenes involved in multiple KEGG pathways or with higher absolute value of the log2 fold change. Overall, we identified seven unigenes, tumor necrosis factor receptor superfamily member 13B (*TNFRSF13B*), C–C motif chemokine 4-like (*CCL4*), C–X–C chemokine receptor type 4 (*CXCR4*), interleukin 2 receptor alpha (*IL2RA*), *MHC* class I heavy chain (*MHCIα*), transporter of antigen processing 2 (*TAP2*) and immunoglobulin alpha heavy chain (*IgH*), that have been reported to play important roles in the immune response. Among these seven unigenes, only three genes (*IgH*, *MHCIα*, and *IL2RA*) have been identified in goose, and the complete coding sequences (CDs) of *IgH* and *MHCIα* have not yet been recorded in NCBI database. Based on their predicted functions, the seven unigenes belong to three functional groups which are cytokine and cytokine receptor, antigen processing and presentation as well as humoral immune effectors, respectively.

**Table 4:** Differential expression of genes involved in the immune response.

Unigene ID	Gene Symbol	Gene Description	Organism	log2 Fold Change	Pathway
CL22500Contig1	*IgH*	immunoglobulin alpha heavy chain	*Anser anser domesticus*	8.01485715881499	HCL, NKC, BCR, *Fc*ε*RI*, FcγR, IIN
CL36733Contig1	*CXCR4*	C–X–C chemokine receptor type 4	*Melopsittacus undulatus*	2.82461947963658	CSP, LTM, IIN
CL28326Contig1	*CXCR6*	C–X–C chemokine receptor type 6	*Anas platyrhynchos*	2.26986828363545	CSP
CL36371Contig1	*XCR1*	chemokine XC receptor 1	*Anas platyrhynchos*	1.9920108201249	CSP, CCR
CL35006Contig1	*CCL19*	chemokine C–C motif ligand 19 precursor	*Anas platyrhynchos*	1.87882406908735	CSP
CL34468Contig1	*CCL4*	c-C motif chemokine 4-like	*Anas platyrhynchos*	1.87191382409167	CSP, TLR, CDP, CCR
CL45402Contig1	*IgH*	immunoglobulin alpha heavy chain	*Anser anser domesticus*	1.86841583899162	HCL, NKC, BCR, *Fc*ε*RI*, FcγR, IIN
CL46049Contig1	*CCL26*	c-C motif chemokine 26-like	*Meleagris gallopavo*	1.80369233288299	CSP
CL13415Contig1	*PLCG2*	1-phosphatidylinositol 4,5-bisphosphate phosphodiesterase gamma-2 isoform X4	*Gallus gallus*	1.72847057141819	NKC, BCR, *Fc*ε*RI*, FcγR, LTM
CL36217Contig1	*OPN*	osteopontin	*Anas platyrhynchos*	1.723521984199	TLR
comp93267_c1_seq1	*MHCIα*	MHC class I heavy chain	*Anser anser*	1.55884992861107	APP
CL45933Contig1	*TAP2*	transporter of antigen processing 2	*Anas platyrhynchos*	1.18018757654374	APP
CL6275Contig1	*IL2RA*	interleukin 2 receptor alpha	*Anser cygnoides*	1.17080106279218	HCL, CCR
CL15887Contig1	*IL21R*	interleukin-21 receptor isoform X1	*Anas platyrhynchos*	1.09522512352901	CCR
CL7617Contig1	*M3K8*	mitogen-activated protein kinase kinase kinase 8 isoform X2	*Anas platyrhynchos*	1.04305025312537	TLR, TCR
CL47027Contig1	*MASP1*	mannan-binding lectin serine protease 1 isoform X1	*Columba livia*	–	CCC
				3.10860084300754	
CL12315Contig1	*IL8*	interleukin-8-like	*Anas platyrhynchos*	–	CSP, TLR, NODLR, RIGLR, CCR
				2.12963662747173	
CL36341Contig1	*CSF3R*	colony stimulating factor 3 receptor (granulocyte)	*Anas platyrhynchos*	–	HCL
				1.92500564529962	
CL1203Contig2	*TNFRSF13B*	tumor necrosis factor receptor superfamily member 13B	*Anas platyrhynchos*	–	IIN
				1.36191602157385	
CL20554Contig1	*ITB3*	integrin beta-3-like	*Anas platyrhynchos*	–	HCL
				1.34147190439098	
CL519Contig2	*TPA*	tissue-type plasminogen activator	*Anas platyrhynchos*	–	CCC
				1.27377380687262	
CL45872Contig1	*MP2K3*	dual specificity mitogen-activated protein kinase kinase 3	*Columba livia*	–	TLR, *Fc*ε*RI*
				1.02391521347926	

Abbreviation for pathways CSP: Chemokine signaling pathway; CCC: Complement and coagulation cascades; APP: Antigen processing and presentation; TLR: Toll-like receptor signaling pathway; NODLR: NOD-like receptor signaling pathway; RIGLR: RIG-I-like receptor signaling pathway; CDP: Cytosolic DNA-sensing pathway; HCL: Hematopoietic cell lineage; NKC: Natural killer cell mediated cytotoxicity; TCR: T cell receptor signaling pathway; BCR: B cell receptor signaling pathway; *FcεRI*: Fc epsilon RI signaling pathway; FcγR: Fc gamma R-mediated phagocytosis; LTM: Leukocyte transendothelial migration; IIN: Intestinal immune network for IgA production; CCR: Cytokine–cytokine receptor interaction.

#### 2.6.1. Cytokines and Cytokine Receptors

In the cytokine and cytokine receptor group, the interaction between a cytokine and its receptor can mediate the cytokines signaling pathway which is regarded as a cascade amplifier. Generally speaking, many cytokines will not directly result in biological action. However, the interaction between a cytokine and its receptor plays a pivotal role in cascades, which can trigger other downstream factors and then result in biological action. The interaction between cytokines and cytokine receptors is shown in Figure S1; the green boxes indicate that the unigenes were identified in our study.

*TNFRSF13B* (also known as transmembrane activator and calcium-modulating and cyclophilin ligand interactor, or *TACI*) served as the shared receptor of B-cell activating factor (*BAFF*) and a proliferation inducting ligand (*APRIL*), which is expressed on the surface of B-lymphocytes. In the present study, the *TNFRSF13B* expression level of goslings was higher than that of adult geese, which was inconsistent with lower expression level in neonatal humans and mice [35]. There is not sufficient evidence to explain the discrepancy between goose and mammal. However, it has been reported that *TACI* as an inhibitory *BAFF* receptor can inhibit the proliferation of B cells [36]. We suspected that high expression of *TACI* in goslings may excessively inhibit the proliferation of B cells, thereby resulting in the deficiency of humoral immunity.

*CXCR4* is expressed on B cells at various stages and in cells of hematopoietic lineages [37]. Some studies have demonstrated its crucial role in the localization of plasma cells in spleen. The retention of developing B cells in the bone marrow also requires CXCR4 and its ligand C–X–C chemokine 12-like (*CXCL12*) [38]. Using suppressive subtractive hybridization (SSH), Vanderven, *et al.* described that *CXCR4* transcripts were enriched in duck lung at 1 dpi with low pathogenic avian influenza virus [39]. However, whether *CXCR4* participates in the restriction of low pathogenic avian influenza virus requires further investigations.

Another member of this group is *CCL4* which is the homolog of macrophage inflammatory protein-1β (*MIP-1β*) in human. *CCL4* as an immune activation gene was expressed in activated macrophages, lymphocytes and fibroblasts. Once produced, it will induce the expression of its receptor C–C chemokine receptor type 5-like (*CCR5*) on immune cells, thereby inducing the accumulation of immune cells at the infection sites.

In human, *IL2RA* (also known as *CD25*), as a feature of CD4^+^CD25^+^ T cells, has been investigated widely in autoimmune diseases. In the study of avian *IL2RA*, it was reported that CD25^+^ cells were up-regulated dramatically in avians with H9N2 virus infection, which indicated that IL2RA is closely connected with the infection of H9N2 virus [40,41]. CD4^+^CD25^+^ T cells, as one type of Treg cell, can suppress the proliferation and cytokine secretion by other T cell populations [42] to achieve a balance between positive and negative immune responses. In our study, the *IL2RA* transcript in spleens of adult geese was increased in comparison to goslings, indicating that CD4^+^CD25^+^ T cells might contribute to controlling the excessive immune response in adult geese. Currently, the function of goose *CD25* remains unclear and requires further investigation.

#### 2.6.2. Antigen Processing and Presentation

In the antigen processing and presentation group, we selected *MHCIα* and *TAP2*. Both of them are involved in endogenous antigen processing and presentation. *TAP2* is the one component of *TAP* heterodimer, while another subunit of *TAP* protein is *TAP1*. Furthermore, *MHCIα* is the heavy chain of MHC class I which consists of the transmembrane α chain and a soluble β2-microglobulin. In the antigen processing and presentation pathway, endogenous antigens are proteolytically degraded into cytosolic peptides. With the presence of *TAP* heterodimer in the endoplasmic reticulum membrane (ER), these cytosolic peptides can be delivered into the ER where they bind to the Class I molecules of *MHC* to form *MHC I*-peptide complexes. Subsequently, these complexes are secreted from the ER and processed in the Golgi apparatus. Finally, they are presented on the cell surface to induce the specific immune activity of cytotoxic T lymphocytes (CTLs) [43]. In the adult goose, the increased expression of *MHCI* and *TAP2* would generally result in that the presentation of more virus epitopes presented to CTLs, which would presumably increase the efficiency of killing the infected cells.

The virus might be immediately cleared from host cells upon invasion, such that adult geese have low susceptibility to pathogens when compared to neonatal birds.

#### 2.6.3. Humoral Immune Effectors

Within the humoral immune effectors group, only *IgH* was identified. In general, *IgH* is expressed in parallel with Immunoglobulin light chain (*IgL*), but we only found *IgH* to be markedly up-regulated in adult goose. It is possible that many reads from the *IgL* gene may be removed from adult goose transcript data, resulting in comparable expression levels throughout goose development. In short, the expression of immunoglobulin was increased dramatically in adult goose, which may be caused by the development of the immune system. With developed humoral immunity in adult geese, a more robust immune response to pathogens was initiated compared with that in goslings. Further, more immunoglobulin in adult geese would neutralize a virus, thereby contributing to virus resistance. Therefore, adult geese show higher diseases resistance than do goslings.

**Figure 9:**
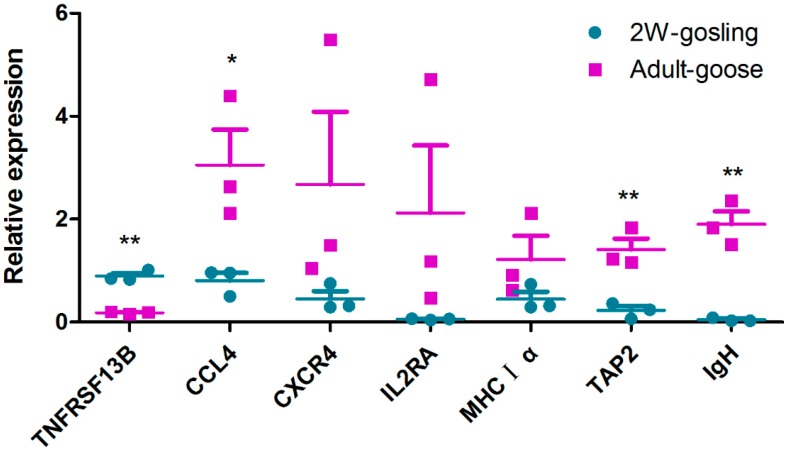
Validation of the gene expression profile by qRT-PCR. Each dot represents an individual goose in one of the two groups (one group is two-week-old goslings; the other group is adult geese). The mRNA level of each spleen sample was quantified by qRT-PCR in triplicate for each gene. *GAPDH* was amplified as an internal control. The chart was constructed in GraphPad Prism 5. The relative expression levels were compared using multiple *t*-tests. Data are represented as the mean ± SEM (*n* = 3). For gene symbols representing different genes, refer to Table 4. * indicates significant differences (*p* ≤ 0.05), ** indicates extremely significant differences (*p* ≤ 0.01).

Finally, to verify the reliability and validity of the transcriptome data, the expression of these candidate genes were detected in three individuals of each group by qRT-PCR. As shown in Figure 9, compared to goslings, *CXCR4*, *MHCIα* and *IL2RA* were up-regulated in adult geese. The expression of *CCL4* was significantly up-regulated (*p* < 0.05) in adult geese compared to that in goslings. Furthermore, *IgH* and *TAP2* were extremely significantly up-regulated (*p* ≤ 0.01) in adult bird. However, *TNFRSF13B* was extremely significantly down-regulated (*p* ≤ 0.01) in adult geese in comparison with goslings. In summary, the expression levels of all the candidate unigenes were consistent with our transcriptome results, which suggest that these genes are truly differentially expressed between adults and goslings.

## 3. Materials and Methods

### 3.1. Ethics Statement

The animal studies were approved by the Institutional Animal Care and Use Committee of Sichuan Agriculture University (No. XF2014-18), Sichuan, China and followed National Institutes of Health guidelines for the performance of animal experiments.

### 3.2. Sample, H&E Staining and cDNA Library Preparation

All geese purchased from the waterfowl breeding center of Sichuan Agriculture University. Geese were housed individually and provided with sufficient feed, vegetables and water *ad libitum*. One two-week-old gosling and one adult goose were euthanized to collect spleen samples. One part of each of spleen sample was fixed in Bouin’s fluid, after which the samples were dehydrated in graded alcohol, cleared in xylene and then embedded into paraffin. Finally, the sections were cut into 6-µm thick slices and stained with hematoxylin and eosin (H&E).

The remaining spleen samples were rapidly removed and kept in liquid nitrogen for extracting RNA. Total RNA was extracted using Trizol reagent (Invitrogen, Carlsbad, CA, USA) following the manufacturer’s protocol, and the remnant DNA was digested by DNase. The quality of extracted RNA was checked using a NanoDrop and Agilent 2100 (Agilent Technologies, Santa Clara, CA, USA). Using the TruSeq_RNA_Sample Preparation Kit_v2 (Ilumina, San Diego, CA, USA), we created a cDNA library. Briefly, total RNA was purified by oligo (dT) magnetic beads to obtain mRNA. Fragmentation buffer was used to fragment the mRNA into short fragments. Using these short fragments as templates, the double-stranded cDNA was synthesized using random hexamer primers. Then, they were purified for terminal repair, poly (A) addition and the ligation of sequencing adapters. Finally, we created the cDNA library by amplifying the fragments.

### 3.3. Quality Assessment and de Novo Assembly

The fragments in cDNA library were tested using an Agilent 2100 Bioanalyzer (Agilent Technologies, Santa Clara, CA, USA), and only the fragments in line with quality standards were then sequenced on an Illumina HiSeq™ 2000. To verify the quality of raw data, the raw data were evaluated using FastQC and the low-quality reads were removed to gain clean reads through the NGS QC Toolkit (Section 2.3) [44]. To detect whether the samples were clean, 50 million reads were selected randomly to BLAST against the Nr database with a cut-off *E*-value of 10^−1^ and the coverage above 80%. In addition these clean reads of two samples were *de novo* spliced by Trinity software [12]. To generate effective unigenes, the TGICL package [13], with the default parameter values, was used to exclude redundant data. Finally, the unigenes were generated.

### 3.4. Functional Annotation and Classification

To identify the function of the unigenes, according to the BLAST algorithm published by Altschul, *et al.* [45], all assembled unigenes were searched for the homologous sequences using the BLASTX program against public protein databases (Nr, Swiss-Prot, GO, KOG, KEGG) with a cut-off *E*-value of 10^−5^. To further evaluate the completeness of our transcriptome data and continue to perfect our annotation process, unigenes were searched for the genes involved in KOG classifications [46]. In addition, the GO database, an international standardized gene functional classification system, was used to functionally classify unigenes with the most significant BLASTX hits against Nr database [47]. Using GO ID, unigenes were mapped to GO terms belonging to various categories (biological process, cellular component and molecular function). Finally, to identify the biological pathways active in the goose and further understand their biological functions and the interactions among genes, all annotated unigenes were subjected to a search against the KEGG database [48].

### 3.5. Differential Expression Analysis and Enrichment Analysis

Using Bowtie 2 [32] and eXpress [33], the clean reads were mapped back to unigenes to calculate the expression of each unigene in each sample. According to the negative binomial distribution test in the DESeq software package [49], the number of reads mapped to the each gene in proper pairs was calculated and normalized by FPKM [50] method, which accounts for the influence of the different lengths and sequcencing depth among genes on the calculation of fragments. Finally, the expression of each unigene in each sample was evaluated by base mean value. To identify the transcripts with differential expression between gosling and adult goose, the FDR was used to adjust the threshold of the *p* value in multiple hypothesis testing. *p* ≤ 0.05 was used as the significance threshold for gene expression differences. That the expression level of a gene in the adults was higher than that of goslings denoted the gene was up-regulated, or *vice versa*. GO and KEGG pathway enrichment analyses were performed to identify the pathways in which the discrepant unigenes participated, while the hypergeometric distribution test was used to determine the pathways significantly enriched in differentially expressed genes.

### 3.6. Identification of Candidate Genes and qRT-PCR Validation

Among the annotated unigenes that were involved in the 16 immune system KEGG pathways, seven genes that were differentially expressed at different developmental stages were selected and confirmed by qRT-PCR. The primers of the seven immune-relevant genes were designed for qRT-PCR analysis according to the sequences from our transcriptome data, and they are listed in Table S1. qRT-PCR analysis was used to validate the reliability of the Illumina Sequencing. Three individuals from each group (two-week-old goslings and adult geese) were euthanatized for the collection of spleen samples. Total RNA was extracted as described for the cDNA library preparation and then reverse transcribed using EvaGreen 2× qPCR MasterMix (Abm, Milton, ON, Canada). The qRT-PCR analysis was performed by QuantiFast SYBR Green PCR Kit (QIAGEN, Dusseldorf, Germany) and the Bio-Rad CFX96 Real-Time Detection System (Bio-Rad, Hercules, CA, USA). The goose *GAPDH* was chosen as the internal control gene and the negative control used no cDNA template. The differential mRNA expression levels of the seven genes were verified in triplicate.

## 4. Conclusions

In the present study, histological analysis comparing gosling and adult goose was performed and well-developed spleen parenchyma was detected in the adult goose. Moreover, we have conducted an Illumina sequencing project on goose spleens to screen for immune-related genes. Here, 50,390 unigenes are identified, among which there were 558 important immune-related genes and 2700 differentially expressed genes. Among these differential expression genes, 22 unigenes were involved in the immune response, including 15 up-regulated unigenes and seven down-regulated unigenes.

It is also noteworthy that 23 predicted cytokines were identified and compared with the homolog of human, chicken and duck, which may facilitate goose molecular immune research and vaccine development. Finally, seven genes were assessed using qRT-PCR to verify the reliability and validity of the transcriptome data. Overall, these data will accelerate our understanding of the goose immune system and the immune development of waterfowl.

## Supplementary Materials

Supplementary materials can be found at http://www.mdpi.com/1422-0067/16/09/22904/s1.
